# Fine structural dynamics showing functional changes of anterior pituitary cells in the rat

**Published:** 2004-06-01

**Authors:** Kazumasa Kurosumi

**Affiliations:** Professor Emeritus, Gunma University, 3-39-15, Showa-machi, Maebashi, Gunma 371-8512

**Keywords:** Electron microscopy, anterior pituitary, rat, cell types, exocytosis, immunocytochemistry

## Abstract

The cell types in the rat anterior pituitary were classified according to the function of hormone produced by each type cells. Depending upon the age and sex of the rat studied, the fine structure of the cell remarkably differs from each other. The name of cell type indicates the name (abbreviation) of hormone produced by each type cell, which is also divided into two or three subtypes according to the ultrastructure or developing stage viz. immature, intermediate and mature types. The immunocytochemistry is a useful method both for light and electron microscopy in order to study the activity of every cell organelles, because it is easy to indicate the localization of hormones in the cell. The anterior pituitary hormones are all proteins or polypeptides fragmented from the former, and making antibodies of these hormones is rather easy. Significance of every cell organelles in the endocrine function was clarified.

## Introduction

The rat anterior pituitary is very frequently used for the study in cell biology of secretion mechanism of hormonal proteins, because there is a control mechanism of hormone production by the so-called feed-back mechanism, for example the increase of thyroid hormone circulating in blood stream may inhibit the production of thyroid-stimulating hormone from the anterior pituitary. A similar but reverse reaction may occur as a stimulation of gonadotropic cells of the anterior pituitary after castration that is the ablation of gonads which secrete sexual hormones. Such physiological experiments changing of peripheral hormone content may alter the production rate of higher controlling hormone produced by the hypothalamus and anterior pituitary. Such routine experiments of endocrinology may be studied for clarification of activity of intracellular organelles and stockyard of products, that is the secretory granules in the endocrine cells. In such experimental studies, immunocytochemical technique is useful for demonstration of the intracellular place of production and storage of hormones.

## Classification of cell types and its functional significance

By light microscopy, the anterior pituitary cells were classified into three different types according to their staining behavior to dies; acidophil (red in Azan staining), basophil (blue) and chromophobe (colorless). Such staining reactions to dies are the chemical difference of secretory protein which are hormone or precursor of hormones. Acidophils contain simple protein, while basophils contain glycoprotein and chromophobes are either hormone-producing or non-producing cells. These two types of the chromophobes are large chromophobes and small chromophobes, the former produces ACTH that is the main product and its precursor is known as POMC (pro-opiomelanocortin) that is a large protein but its fission produces different polipeptides, endorphine, MSH (melanophore-stimulating hormone) and ACTH (adrenocorticotropic hormone). The second chromophobe is non-secretory but supporting in function and called folliculo-stellate cells from their shape as observed with the electron microscope. This cell type was reported to contain S-100 protein and fibronectin as shown by light microscope immunohistochemistry.

Acidophils stained red in Azan staining are also classified into two types, one produces GH (growth hormone) and called somatotroph, and the second produces PRL (prolactin) whose producer is called mammotroph or lactotroph, because this cell stimulates the mammary glands in pregnant female by which milk is secreted. The basophils contain glycoprotein hormones and therefore intense positive in PAS reaction which stains glucose and similar carbohydrate. They stain blue or purple with Azan staining. TSH (thyroid-stimulating hormone), LH (luteinizing hormone) and FSH (follicle-stimulating hormone) are all glycoprotein hormones. LH and FSH are called together gonadotropic hormones. The producer cells of this gonadotropic hormones are called gonadotrophs. While TSH (thyroid-stimulating hormone or thyrotropin) is produced by TSH cells or thyrotrophs. For the nomenclature of anterior pituitary cells Greek alphabet was used by Romeis,1) and such Greek names were also used in electron microscopic studies.2),3) Because different authors called the same cells by different names, a confusion occurs, then no more researchers used Greek alphabet for anterior pituitary cells but they called by hormone names (abbreviations). For names of subtypes numerals indicating the order of discovery, the Type I, II, III were used. These nomenclature with numerals changed to those indicating the fine structure or size (maturity) of secretory granules, viz., immature, intermediate and mature types (Table I).

**Fig. 1:**
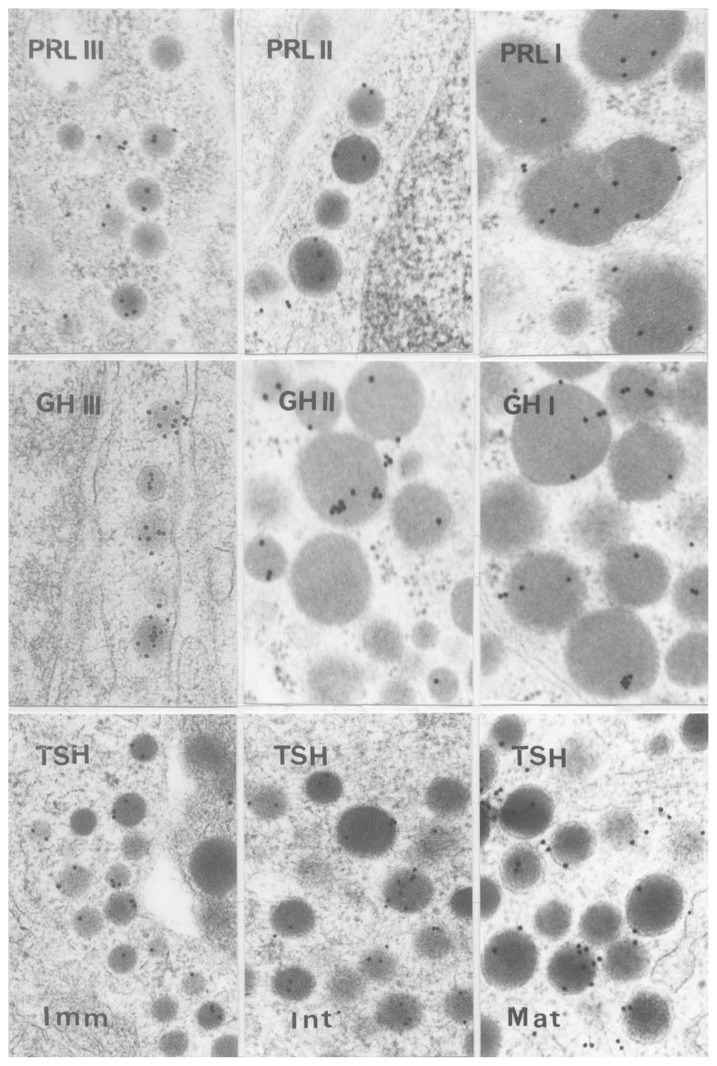
Immunoelectron micrographs showing secretory granules of various cells of the rat anterior pituitary. The cell types of each group are immature (Type III), intermediate (Type II), and mature (Type I) from left to right. PRL: prolactin cells, GH: growth hormone cells, TSH: thyrotropin cells. × 50,000.

Secretory granules of these three types are shown in Fig. 1. Type I of PRL cell was first discovered and known to be characterized by large irregular secretory granules.4) This type of PRL cell depends on the female hormone, that is estrogen,5) the Type II PRL cell contains middle-sized round granules and the last found PRL cells contain small round granules and called Type III.6) Because the small granule cells were found in young rat pituitary, this type was renamed to immature type. GH cells are the same in naming of subtypes as those of PRL cells. Granules of GH cells discovered first were large round and mainly found in male rat pituitary. The Type II GH cells contain mixed small and large granules and found in female rat pituitary, and the last found GH cells possess small granules similar to PRL cells and initially called Type III but renamed to immature type,7) while Type II was changed to intermediate type and then Type I became to mature type.

TSH cells were studied by Ozawa and Kurosumi8) and no name of types with numerals were used, but they were classified into immature, intermediate and mature type from the beginning of the study (Fig. 1).

**Fig. 2:**
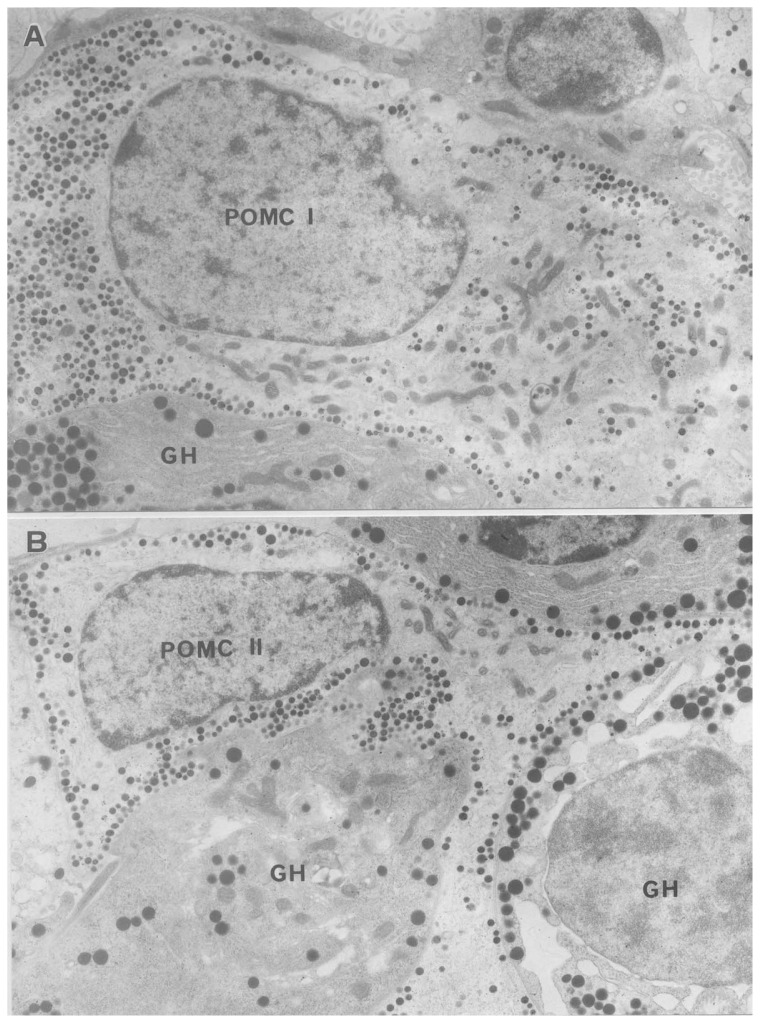
POMC (pro-opiomelanocortin) cells of the anterior pituitary of a young adult rat identified by immunoelectron microscopic reaction using anti-ACTH. **A**: Type I POMC cell (intermediate type) × 9,000. **B**: Type II POMC cell (mature type). This cell is irregular in shape, extend long processes and surrounded by GH cells. × 8,000.

**Fig. 3:**
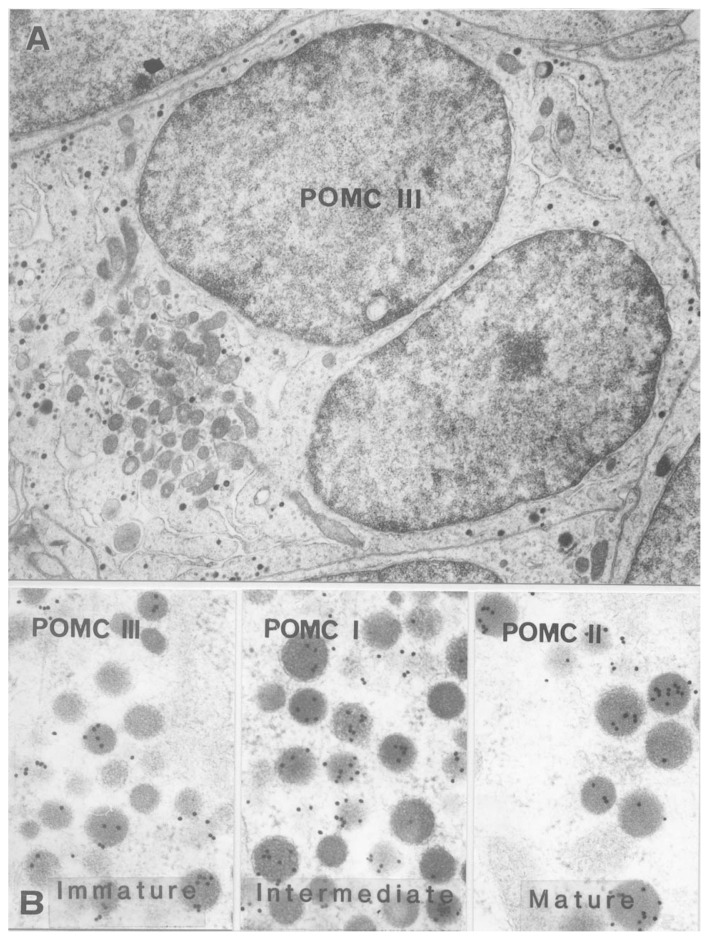
**A**: POMC cell of immature type (Type III). The cell contains two nuclei and a small amount of cytoplasm including a heap of mitochondria near the nuclei. Secretory granules are very small in size. This micrograph was taken from a young rat. × 10,000. **B**: Secretory granules reacted with anti-ACTH serum. From left to right, immature (Type III), intermediate (Type I) and mature (Type II) cells. × 50,000.

As to the renaming, POMC cells are unique, because the first type of POMC (ACTH) cell was described by Kurosumi and Kobayashi9) therefore it was named Type I, but it was renamed to the intermediate type according to the granule size (Figs. 2 and 3). After we described this type as ACTH cell, some American researchers10)–12) reported another type of POMC (ACTH) cells which are star-like in shape and granules are arranged in a row just beneath the cell surface (Fig. 2). This type was called Type II and later mature type,13) and Type III which was later called immature type was found14) (Fig. 3).

The basophils producing glycoprotein hormones, especially gonadotrophs are slightly different from the formerly described cell types. Their homones are composed of alpha and beta subunits. The chemical structure of alpha subunit is common among TSH, LH, and FSH, but those of beta subunits are different from each other, and hence antibodies for each beta subunits were made for the immunocytochemistry. Morphologically gonadotrophs are divided into two types; one was called FSH cell and another LH cell described in an old literature.15) The former contains large and small secretory granules, while the latter cell contains only small granules, and orderly called Type I and II gonadotrophs. The immunoelectron microscopy of Type I gonadotroph was performed with ferritin particles as the marker. LH beta immuno-labelling was seen on both the small and large secretory granules, while the marker for FSH beta subunits was only found on the large secretory granules.16) It may be concluded that the Type I gonadotroph has small and large granules, and hence Type I cells produce both LH and FSH, while Type II cells only contain LH. The fact was reported that FSH reduces when animals getting older, as occurrence of Type I gonadotroph decreases in number.17)

## Endoplasmic reticulum and protein hormone synthesis

Rough-surfaced endoplasmic reticulum is well known to be highly related to the synthesis of protein. Because the anterior pituitary cells contain well developed endoplasmic reticulum of this type, these cells produce a great amount of protein hormones. The endoplasmic reticulum consists of sac-like structures which are called cisternae, and the outer surface of the membrane outlining the cisternae many small granules are scattered. They are called ribosomes, because they contain RNA. Amino acids trapped into the cytoplasm are transported by messenger RNA to the ribosomes where joined to each other and become the polypeptide, in which the sequence of amino acids is coded in DNA in the nucleus as the gene information. The newly synthesized protein or polypeptide at the membrane-bound ribosomes are transfered into the cavity of the endoplasmic reticulum passing through a narrow channel penetrating the membrane of the cisternae. As testosterone is known to hinder the release of gonadotropin, it was applied to the castrated male in which Type I gonadotrophs were stimulated and LH is highly concentrated in the cisternae and observed as very dark substance accumulated in the rough endoplasmic reticulum which is positive to anti-LH labeled with gold particles.18) Cryoultrathin section showed positive reaction in the cavity of the rough endoplasmic reticulum in a study using anti-serum against FSH beta subunit. Round granules appear in the cisterna expanded with liquid substance in TSH cells after thyroidectomy and such intracisternal granules are also immunoreactive with anti-TSH.19)

## Golgi apparatus and its role in the formation of secretory granules

As observed by light microscopy, a specific net-like structure blackened by the impregnation with heavy metal such as silver or osmium was discovered by Camillo Golgi in 189820) and named internal reticular apparatus. This cell organelle is now called the Golgi apparatus or Golgi complex. Biological terms either Golgi bodies or dictyosomes are not correct for this cell organelle in the mammalian cells, because these terms signify scattered bodies occurring in the cells of lower animals or plants.21) Electron microscopist who first observed Golgi apparatus was Dalton.22) Then Dalton and Felix23) reported the fine structure of Golgi apparatus in detail: three components are vacuoles, lamellae and vesicles, and they called Golgi complex altogether. Function of Golgi apparatus in secretory cells is formation of secretory granules. The main part of Golgi apparatus is the parallel lamellae, that is composed of flattened sacs which are piled up one another, forming parallel lamellae, that is curved and hence convex side and concave side are distinguished, and the convex side is called cis-side and the concave side is called transside. 24) Secretory substance is synthesized at the ribosomes and stored in the cavity of rough endoplasmic reticulum, then transmitted to the cis-side of Golgi lamellae through the transitional elements which consists of small process extending from the outer surface of rough endoplasmic reticulum, which may be pinched off becoming vesicles moving to the Golgi apparatus and finally fuse to the sacs of Golgi lamellae transporting the material in the vesicles.

**Fig. 4:**
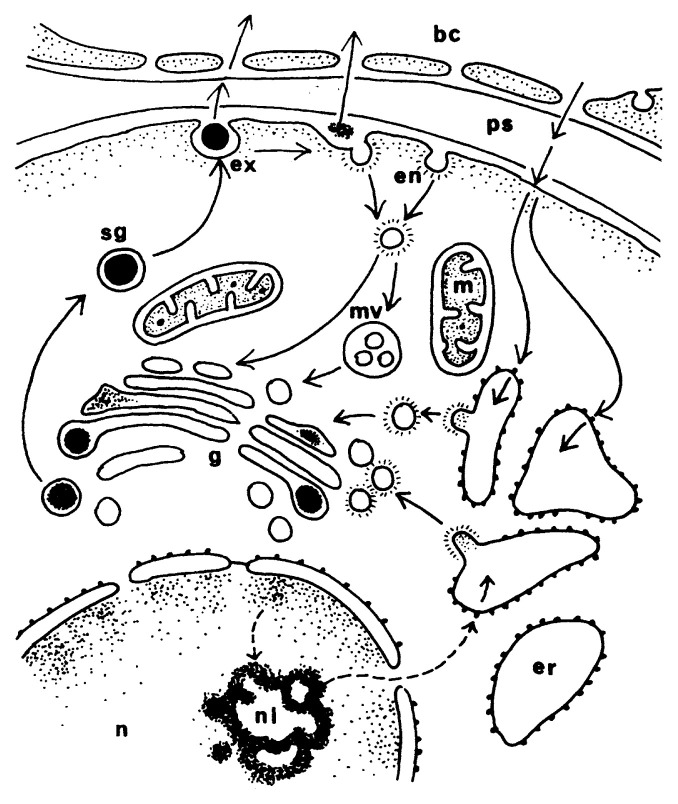
Diagram of peptide hormone secreting anterior pituitary cell. Solid line with arrows show the route of moving secretory substance and its materials. Broken line with arrows shows the route of information of genes and mRNA for hormone synthesis. **bc**: blood capillary, **ps**: pericapillary space, **n**: nucleus, **nl**: nucleolus, **er**: rough endoplasmic reticulum, **m**: mitochondria, **g**: Golgi apparatus, **sg**: secretory granule, **ex**: exocytosis, **en**: endocytosis, **mv**: multivesicular body (lysosome).

In ordinary electron micrographs secretory substance is seen very dark and easily found in these pictures. However, hormonal identification is not easy because for anti-serum of hormone to penetrate into plastic embedded sections is very difficult. To avoid such a physical hinder, we used ultracryo-microtomy without embedding and the gold labeled anti-serum easily enters into the cryosection without plastic embedding medium around the biological specimen, and therefore serum with gold label can approach the hormone in the tissue. As seen in the diagram (Fig. 4) presence of PRL in the Golgi lamellae as well as in the secretory granules is eassily found by the immunoreactivity of gold labeled antiserum. The condensation of secretory substance which is packed into vacuoles produces secretory granules. A similar event may occur for the formation of lysosomes. The main substance in the lysosomes are hydrolytic enzymes which are also protein synthesized at the rough endoplasmic reticulum, transported to Golgi apparatus just like the secretory protein. Acid phosphatase is one of typical marker enzymes of the lysosome and histochemically demonstrated shown in the diagram as a multivesicular body (mv) and in GERL25) or trans Golgi network.26)

Processing and sorting of secretory substance may occur simultaneously both in the Golgi stack (lamellae) and in the GERL (trans Golgi network) (Fig. 4). Chemical processing that is the fission of the large molecule in the POMC cells producing small peptide hormons may begin at the Golgi lamellae but such chemical events may continue during the movement of secretory granules from Golgi area to the cell surface.

## Colocalization of multiple hormones and other substance in a single secretory granule and transport of granules

Secretory granules are variable in size, shape, densities as well as their content. Some anterior pituitary cells contain two or more types of secretory granules in a single cell, for example Type I gonadotroph contains large and small secretory granules, and two different hormones, that is LH and FSH. LH is found in both small and large granules, but FSH is found only in large granules.16) Large granules are generally low in density and hence they look rather light.

GH and PRL are similar in chemical structure; they are both simple protein and stained both acidophilic. These two hormones are usually found in different cell types in the rat. Fumagalli and Zanini27) found colocalization of GH and PRL in the same granules or in different granules in the same cell in female bovine pituitary. Nikitovitch-Winer *et al*.28) reported mammosomatotrophs containing GH and PRL in the normal rats. Colocalization of GH and PRL was also reported in the same cell in a pregnant rat.29) Because GH and PRL are similar in chemical structure hence some variations may occur in genetic information that is a kind of malformation producing these two hormones in the same single cell. Other substances than the hormones but related in function of secretory granule fomation may colocalize with the normal hormone such as PRL.30) They belong to carrier substance known as granin family that is chromogranin A, secretogranin I (=chromogranin B) and secretogranin II (= chromogranin C). It is known that these proteins belonging to the granin family are effective in aggregation of hormonal proteins packaging into the secretory granules.31) Type I PRL cell is the only one cell type having irregularly shaped secretory granules, which may be formed by fusion of small round granules. Granin family substances may be useful for aggregation of precursor small granules of PRL, forming irregularly shaped granules.

On the other hand, the case in POMC cells is reverse, the processing of POMC is fission of large molecular precursor protein into small peptides such as MSH, ACTH, and endorphin etc. Proteolytic enzymes must colocalize with the precursor POMC and proteolysis may begin in acidic pH condition brought about by the act of proton ATPase in the secretory granules after leaving from the trans Golgi network and begin to move towards the cell periphery.32) In the POMC cells two kinds of secretory granules, dark and clear granules are observed. Immunoelectron microscopy on the cryosections indicated the precursor POMC in the Golgi lamellae, trans Golgi network and dark secretory granules which reside in the Golgi apparatus, while clear secretory granules near the cell periphery may contain mature peptides such as ACTH, JP, MSH derived from POMC after proteolytic processing.33) Therefore, the maturation of secretory granules (from dark to clear granules) and processing of POMC to ACTH and other peptides may occur in the secretory granules during their translocation. The trigger of processing is performed by acidification which activate the proteolysis and these helper substances for the processing are also colocalized in the secretory granules of POMC cells.

**Fig. 5:**
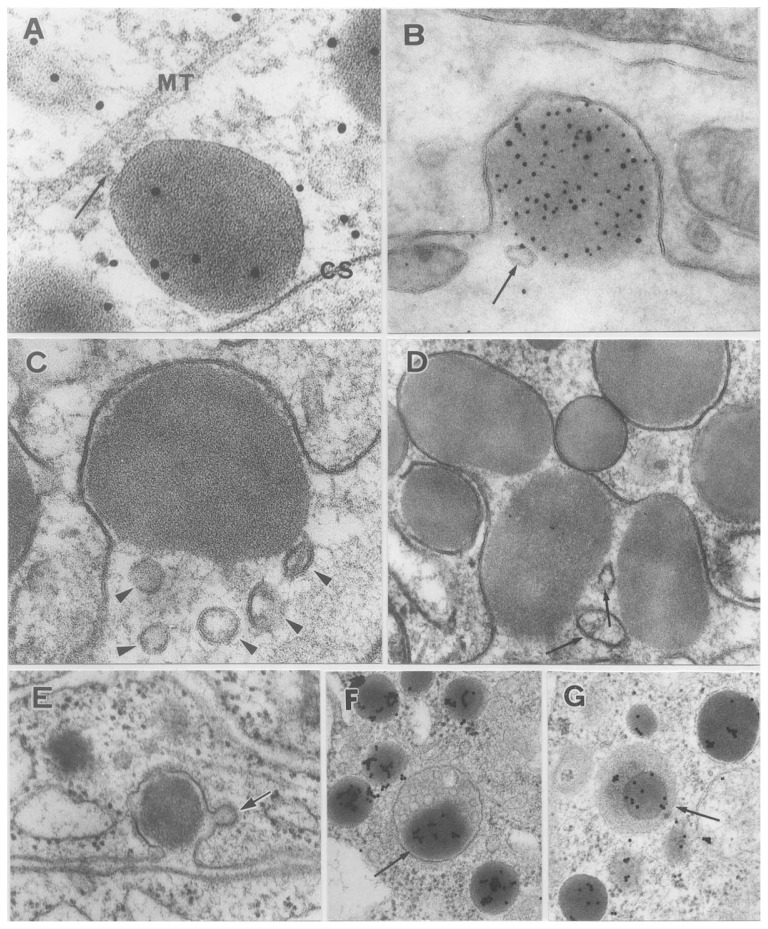
Transport, exocytosis and crinophagy of secretory granules in anterior pituitary cells. **A**: An immunolabeled secretory granule of GH cell is linked to a microtubule (MT) with amorphous substance of unknown nature (arrow). The granule approaches the vicinity of cell surface (CS). × 160,000. **B**: A secretory granule reacted to anti-PRL serum is opened to the extracellular space by the exocytosis. A small vesicle (arrow) is also discharged accompanying with the extruding granule × 120,000. **C**: Exocytosis of a granule of a PRL cell. Small vesicle-like fragments (arrowheads) of cytoplasm are discharged with the secretory granule. × 120,000. **D**: Multigranular exocytosis of PRL cell accompanying with extrusion of small fragments of cytoplasm as shown by small arrows. × 93,000. **E**: Exocytosis and accompanying endocytosis forming a coated vesicle (small arrow). × 44,000. **F** and **G**: Crinophagy of GH cells labeled with gold particles. **F** shows the phagocytosis of a secretory granule by a multi-vesicular body (arrow) × 40,000. In **G** a lysosome engulfed a GH granule (arrow). × 27,000.

For the release of secretory substance, the secretory granules move from the site of production in or near the Golgi apparatus toward the cell surface. In the case of insulin secretion Lacy *et al*.34) advocated a hypothesis that microtubules and contractile microfilaments may participate in the movement of secretory granules. The addition of colchicine to the incubation medium of islet tissue inhibited the secretion of insulin. As seen in Fig. 5A, a secretory granule is inserted between the cell surface and a microtubule and a small mass of unknown substance links the secretory granule to the microtubule, which may act as a guardrail. The unknown substance may role a motor-like action just like kinesin in the case of movement of synaptic vesicles.35)

## Extrusion of secretory granules and associated membrane events

A secretory granule approaches to the cell surface and attaches to the inner aspect of the cell membrane. Fusion between cell membrane and granule membrane occurs at a small point of granule attachment. Then fission of fused membrane takes place. This forms a small pore through which the interior of a granule becomes continuous to the extracellular space. The secretory granule may slip out through the small pore. This mechanism of granule extrusion is now widely called “exocytosis” which was the term proposed by De Duve36) as the antonym of “endocytosis,” the latter covering phagocytosis and pinocytosis. The exocytosis of anterior pituitary granule was first observed by Ichikawa37) and then by Farquhar.38) In normal state most secretory granules are extruded separately and called single exocytosis (Fig. 5B,C), but when the cell is strongly stimulated, many secretory granules are expelled simultaneously and called multigranular exocytosis39) (Fig. 5D).

In order to keep the cell surface area constant, not only discharge of secretory granules, but also other membrane events, such as elimination of membrane material occur along with exocytosis. Exocytosis associated with endocytosis is often observed (Fig. 5E). In this picture a small coated vesicle is formed on the membrane of an extruding secretory granule. Not only membrane material, but also a small amount of extracellular substance is trapped into the cytoplasm with this phenomenon the recycling of membrane material occurs.

**Fig. 6:**
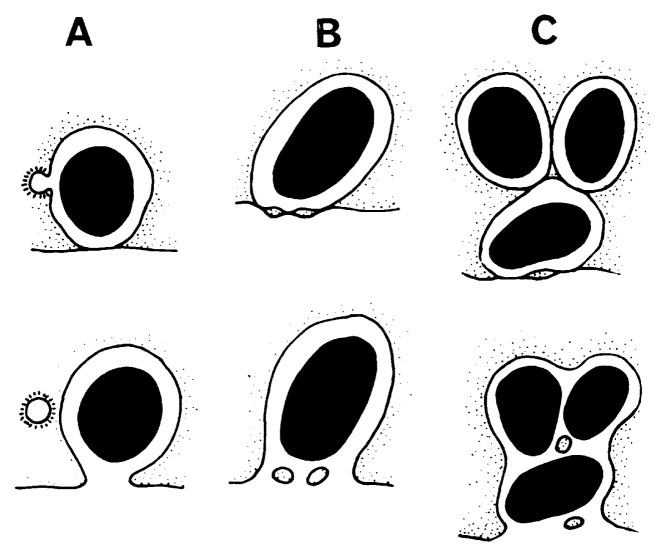
A diagramatic representation of exocytosis-associated membrane events. **A** shows vesicle formation towards the cytoplasm, that is the endocytosis for the membrane retrieval. **B** shows a single exocytosis and **C** represents multigranular exocytosis. In both **B** and **C** small cytoplasmic fragments are discarded to the extracellular space.

As shown in Fig. 5B,C,D, small masses of cytoplasm are detached from the main cell body, and extruded along with secretory granules towards the extracellular space. In this case, small amount of cytoplasmic matrix is discarded, and we call this phenomenon microapocrine secretion. Both the exocytosis associated endocytosis and microapocrine secretion are the same in maintenance of cell membrane material during single or multigranular exocytosis which is a drastic change of cell surface area (Fig. 6).

## Lysosome, multivesicular body and crinophagy

The lysosome may be compared to the digestive tract of the body, because it contains more than a dozen different kinds of digestive enzymes (hydrolases) by which it digest substances engulfed from the outside of the cell, i.e., the last step of phagocytosis and pinocytosis. The lysosomes also digest a part of the cell components, which is called “autophagy.” The optimum pH of hydrolytic enzymes contained in lysosomes are acidic, and acid phosphatase (AcPase) is one of representative enzymes of the lysosome, and therefore AcPase is used for a marker enzyme of the lysosome in both light and electron microscopic histochemistry.

As other protein produced in the cell, enzymes of lysosome are also synthesized in the rough endoplasmic reticulum and transported to the Golgi apparatus. In the Golgi field, the trans-most cisterna which is also called GERL or trans Golgi network, lytic enzymes are sorted from other proteins and packed into vesicles, which are known as primary lysosomes. When phagosomes are fused with primany lysosomes and obtain hydrolases, becoming the secondary lysosome in which the digestion may begin.

The multivesicular bodies are often observed near the Golgi apparatus, i.e., a large vacuole containing many small vesicles (Fig. 5F). The matrix of multivesicular body is positive to AcPase histochemically, but small vesicles inside the body are negative. Therefore, it has been thought that the multivesicular bodies are one kind of lysosomes.40) Smith and Farquhar41) observed the autophagy of secretory granules by lysosomes and multivesicular bodies in PRL cells after weaning of young, and this mechanism is called “crinophagy”.42) (Fig. 5F,G). In these pictures the immunoelectron micrographs showing GH labelling gold particles and dark round bodies in the multivesicular body or lysosome are phagocytosed secretory granules of GH cell. The anterior pituitary works to maintain the production rate constant by controlling the hormone content in the producer cell body by this mechanism.

## Figures and Tables

**Table I:** Cell classification of the rat anterior pituitary.

Classical LM	Functional EM	Subtypes	Secretory granules	Others
Acidophils	Somatotrophs (GH cells)	Immature type (III)	ca 100 nm	rich in young
		Intermed. type (II)	various sizes	rich in female
		Mature type (I)	ca 350 nm	rich in male
	Mammotrophs (PRL cells)	Immature type (III)	ca 100 nm	rich in young
		Intermed. type (II)	various sizes	rich in male
		Mature type (I)	irregular from ca 700 nm	rich in female
Basophils	Thyrotrophs (TSH cells)	Immature type	ca 100 nm	rich in young
		Intermed. type	ca 120 nm	no sex difference
		Mature type	120–180 nm	
	Gonadotrophs (GTH I cells)	Type I	large (ca 500nm) and small (ca 150 nm) (FSH, LH)	rich in male
	Gonadotrophs (GTH II cells)	Type II	only small granule (LH)	rich in female
Chromophobe	Corticotrophs (POMC cells) (or ACTH cells)	Immature type (III)	ca 100 nm	rich in young
		Intermed. type (I)	ca 120 nm	no sex difference
		Mature type (II)	150–200 nm	
	Folliculo-stellate cells	no granule, no hormone secretion contain S-100 protein and fibronectin		

LM: light microscopy, EM: electron microscopy
